# Pallid bands in feathers and associated stable isotope signatures reveal effects of severe weather stressors on fledgling sparrows

**DOI:** 10.7717/peerj.814

**Published:** 2015-03-03

**Authors:** Jeremy D. Ross, Jeffrey F. Kelly, Eli S. Bridge, Michael H. Engel, Dan L. Reinking, W. Alice Boyle

**Affiliations:** 1 Oklahoma Biological Survey, University of Oklahoma, Norman, OK, USA; 2 Department of Biology, University of Oklahoma, Norman, OK, USA; 3 ConocoPhillips School of Geology and Geophysics, University of Oklahoma, Norman, OK, USA; 4 Sutton Avian Research Center, Oklahoma Biological Survey, University of Oklahoma, Bartlesville, OK, USA; 5 Division of Biology, Kansas State University, Manhattan, KS, USA

**Keywords:** disturbance ecology, hail, ground-nesting, stress response, severe storm, fault bars

## Abstract

In August 2013, we observed a high incidence (44%) of synchronous bands of reduced melanin (a type of fault bar we have termed “pallid bands”) across the rectrices of juvenile Grasshopper Sparrows (*Ammodrammus savannarum*) captured near El Reno, Oklahoma. Earlier that year, on May 31, the site was struck by a severe storm which rained hailstones exceeding 5.5 cm diameter and spawned an historic 4.2 km-wide tornado <8 km to the south of the site. We hypothesized that this stressor had induced the pallid bands. An assessment of Grasshopper Sparrow nesting phenology indicated that a large number of nestlings were likely growing tail feathers when the storm hit. The pallid bands were restricted to the distal half of feathers and their widths significantly increased as a function of distance from the tip (i.e., age at formation). We predicted that if stress had caused these pallid bands, then a spike in circulating *δ*^15^N originating from tissue catabolism during the stress response would have been incorporated into the developing feather. From 18 juveniles captured at the site in August we measured *δ*^15^N and *δ*^13^C stable isotope ratios within four to five 0.25–0.40 mg feather sections taken from the distal end of a tail feather; the pallid band, if present, was contained within only one section. After accounting for individual and across-section variation, we found support for our prediction that feather sections containing or located immediately proximal to pallid bands (i.e., the pallid band region) would show significantly higher *δ*^15^N than sections outside this region. In contrast, the feathers of juveniles with pallid bands compared to normal appearing juveniles showed significantly lower *δ*^15^N. A likely explanation is that the latter individuals hatched after the May 31 storm and had consumed a trophically-shifted diet relative to juveniles with pallid bands. Considering this, the juveniles of normal appearance were significantly less abundant within our sample relative to expectations from past cohorts (*z* = − 2.03; *p* = 0.042) and, in as much, suggested widespread nest losses during the storm. Severe weather events may represent major stressors to ground-nesting birds, especially for recent fledglings. We call for others to exploit opportunities to study the effects of severe weather when these rare but devastating stressors impact established field research sites.

## Introduction

**Figure 1:**
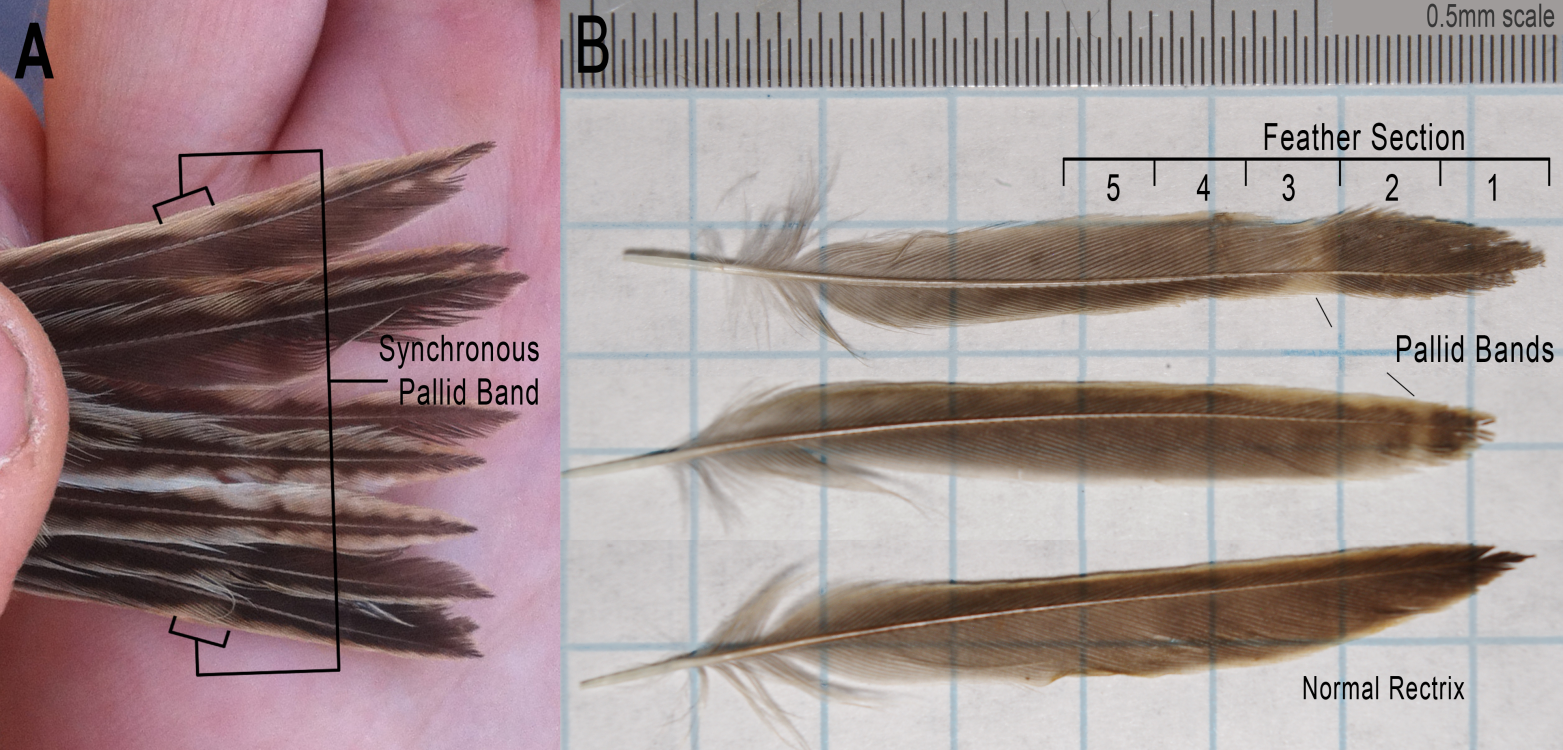
Photographs of Grasshopper Sparrow rectrices from the Grazinglands Research Laboratory. (A) Shows an individual tail with a synchronous pallid band. (B) Illustrates the sections sampled from the rectrices of juvenile Grasshopper Sparrows. Sampling was constrained to provide a minimum of 0.2–0.3 mg from each section. Shown are two examples of feathers with fault bars (top) and one of normal growth (bottom). Photograph 1A by W. Alice Boyle; photograph 1B by Jeremy D. Ross.

Barbule and pigment malformations in feathers customarily described as “fault bars” are generally uncommon in natural bird populations (Riddle, 1908; Michener & Michener, 1938; Wood, 1950; Erritzøe & Busching, 2006; Møller, Erritzøe & Nielsen, 2009). Among 86 European bird species examined by Møller, Erritzøe & Nielsen (2009), the mean incidence of fault bars was only 5.6% and the maximum rate of occurrence for any given species was 38.5%. Furthermore, when fault bars are present they are rarely uniform across all feathers (Bortolotti, Dawson & Murza, 2002; Møller, Erritzøe & Nielsen, 2009), meaning that the probability of fault bars occurring likely varies among feather tracts (Jovani & Blas, 2004; Serrano & Jovani, 2005). In August 2013 we observed an unusually high incidence of apparent fault bars among juvenile Grasshopper Sparrows (*Ammodrammus savannarum*) captured near El Reno, Oklahoma. These individuals had synchronous bands of reduced melanin across the distal half of their retained juvenile rectrices (no freshly-molted tails were observed among the juveniles captured; Fig. 1A). In this species the distal halves of juvenile rectrices grow from mid-way through nestling development through the early post-fledging period (Sutton, 1936; Vickery, 1996; WA Boyle, 2014, unpublished data) and young remain close to their natal site after fledging (Smith, 1963; Hovick et al., 2011). Therefore, the observed feather irregularities in this population likely originated when many offspring were still in the nest or recently fledged.

The meteorological history of the site led us to hypothesize that the high incidence of apparent fault bars could be related to a tornadic thunderstorm that struck the site with large hailstones on May 31, 2013 (Uccellini, 2014). If this were true, then we predicted that the proportion of juveniles captured in August showing these abnormal rectrices would equate to the proportion of an average cohort that would have hatched by May 31. Fortunately, a nesting phenology dataset for Grasshopper Sparrows in Oklahoma had previously been collected by the George M. Sutton Avian Research Center (GMSARC), against which this prediction could be tested. We further hypothesized that the direct and indirect effects of the hailstorm (i.e., impact trauma, abrupt ground-level cooling, and/or reduction in insect prey resources) had been sufficient to induce widespread stress within this population and we predicted that the feather tissue of the apparent fault bars would contain a biochemical signal of this stress response.

Recently-fledged Grasshopper Sparrow young are vulnerable to the stresses of exposure (Hovick et al., 2011). During extremely heavy storms adult birds may flee (Streby et al., 2015) and abandon their nests to the weather (Hanford, 1913; Hume, 1986; Kirkpatrick, Conway & Ali, 2009). Young that survive ‘riding the storm out’ may be traumatized by the event itself or because of reduced provisioning by adults. The developing feathers of young birds can provide indicators of such environmental stressors, including structural and pigmentation deficiencies widely classified as fault bars (Riddle, 1908; Michener & Michener, 1938; Wood, 1950; Erritzøe & Busching, 2006; Møller, Erritzøe & Nielsen, 2009). Fault bars have been attributed to severe fasting (Slagsvold, 1982), habitat degradation (Sodhi, 2002), physical impairments (Møller, 1989), handling by human observers (King & Murphy, 1984; Murphy, Miller & King, 1989; Negro, Bildstein & Bird, 1994), and disease (Romano et al., 2011). These feather malformations may coincide with endogenous spikes of stress hormones in the blood, particularly corticosterone (DesRochers et al., 2009; Lattin et al., 2011; Legagneux et al., 2013; Jenni-Eiermann et al., 2015), and are thought to form because the deposition of keratin and melanin into the growing feather is disrupted (Riddle, 1908; Michener & Michener, 1938; Wood, 1950; Prum & Williamson, 2001; Møller, Erritzøe & Nielsen, 2009).

Generally, fault bars extend symmetrically outward and in a perpendicular direction from the feather vane. Yet, there seem to be two broadly definable morphologies of fault bars that appear in bird feathers: (1) variations of an abrupt region, often narrow (∼1 mm or less), where barbules and sometimes barbs are completely absent (*sensu* Riddle, 1908); versus (2) graded areas of reduced pigmentation/translucence generally >1 mm wide which Michener & Michener (1938) referred to simply as “bands” and Erritzøe & Busching (2006) descriptively referred to (in German) as “Teilleukismus,” roughly translated as “partially-leucistic”. Within a series of papers, James R. King and Mary E. Murphy purported that the abrupt type of fault bar primarily arose after acute mechanical stress, such as when birds were handled, whereas the graded fault bars were induced endogenously, in their case through dietary restriction (King & Murphy, 1984; Murphy, King & Lu, 1988; Murphy, Miller & King, 1989; Murphy & King, 1990). Because unequivocally using the term “fault bar” for such structural and probable causation differences could lead to unnecessary confusion, we have elaborated on Michener & Michener’s (1938) and Erritzøe & Busching’s (2006) terms by referring to the phenomenon observed in this study as a “pallid band.” This term reflects not only the reduced pigmentation in these bands, but it also acknowledges the weakened structure of the associated barbules and distinguishes these feather irregularities from naturally occurring pale bands in feathers. From this point forward, we exclusively refer to the variant of fault bars central to this study as pallid bands.

Along with pallid band inducement, physiological stress can also coincide with shifts in stable isotope ratios (Hobson, Alisauskas & Clark, 1993; Bortolotti et al., 2008; Bortolotti et al., 2009; Hatch, 2012; Fairhurst et al., 2015; though see Polito et al., 2011; Fairhurst et al., 2013). Stress responses in animals often involve muscle catabolism (Buchanan, 2000; Wingfield, 2008), which effectively means the animal is a consumer of itself and is equivalent to it occupying a higher trophic level (Waterlow, 1968; Hobson, Alisauskas & Clark, 1993; Cherel et al., 2005; West et al., 2006). Since the transamination process during protein catabolism leads to an increase in the fractional representation of heavy nitrogen stable isotopes (expressed as *δ*^15^N; Doucett et al., 1999), then either actually or equivalently occupying a higher tropic level should interchangeably result in a detectable increase in *δ*^15^N, especially in high-turnover areas such as the liver or bloodstream (Minagawa & Wada, 1984; Kelly, 2000; Post, 2002; Jardine et al., 2005). In contrast, heavy carbon stable isotopes (*δ*^13^C) should not be affected by tissue catabolism during a stress response, as the underlying carbon base does not change (unlike during an actual trophic progression; Kelly, 2000).

Indeed, during starvation stress events captive Japanese Quail (*Coturnix japonica*) chicks and fasting wild female Ross’s Geese (*Chen rossii*) showed significantly elevated *δ*^15^N but unchanged *δ*^13^C in muscle and liver tissues relative to control groups (Hobson, Alisauskas & Clark, 1993). Similar patterns were reported for fasting penguins (Cherel et al., 2005; though see Polito et al., 2011), seals (Hückstädt et al., 2012), reptiles (McCue & Pollock, 2008), spiders (Oelbermann & Scheu, 2002) and humans (Fuller et al., 2005). In contrast, a restricted though not quite starvation diet was shown to actually reduce *δ*^15^N among nestling seabirds (Williams et al., 2007; Sears, Hatch & O’Brien, 2009) and sparrows (Kempster et al., 2007), and fasting did not induce *δ*^15^N enrichment in whales (Aguilar et al., 2014). Stress-induced elevation of *δ*^15^N is not necessarily limited to starvation events; exposure to toxins without a dietary restriction, for example, can have similar effects (Shaw-Allen et al., 2005; Sanpera et al., 2008).

Nutrients circulating in the blood are incorporated into growing feathers and their stable isotope signatures during growth will be retained as part of the feather until the next molt (Hobson, 1999; Kelly, 2000; West et al., 2006). Since both elevated *δ*^15^N and the formation of pallid bands could be expected to coincide during stress events, we predicted that feather tissues comprising pallid bands would contain spikes in *δ*^15^N. Using single tail feathers sampled from juvenile Grasshopper Sparrows at our study site, we assessed stable isotope patterns at two levels: (1) across sections of individual feathers that contained pallid bands and (2) between juveniles showing pallid bands versus juveniles without pallid bands. With the first comparison, we tested whether the pallid bands were associated with a period of elevated heavy nitrogen consistent with muscle catabolism as part of a stress response. With the second comparison, we determined if there was evidence for trophic successional shifts in isotope ratios indicative of a temporal separation between the groups.

## Methods

### Sample collection and feather measurements

During August 27–28, 2013 we captured juvenile Grasshopper Sparrows (*Ammodrammus savannarum*) by mist-net within a 29.3 ha grassland unit at the United States Department of Agriculture’s Grazinglands Research Laboratory (GRL; N35.555, W98.041) near El Reno, Oklahoma. As part of a standard sequence of morphological measurements, we scored each juvenile’s tail as having pallid bands (evident as reduced pigmentation and/or structural weaknesses; Fig. 1A) or having apparently normal feathers. We photographed the entire tail, removed a single outer rectrix, and stored the feather in a labeled coin envelope.

In the laboratory, we photographed each sampled feather adjacent to a 0.5 mm-scaled ruler, under fixed light sources, and against a white grid paper background. From these pictures we extracted measures of vane length, width of the pallid band, and distance from the pallid band midpoint to the feather tip (all to nearest 0.1 mm) using the program imageJ (Rasband, 2014). Nutritional deficiency can not only induce pallid bands, but also ultimately reduce overall feather growth (Murphy, King & Lu, 1988; Grubb, 2006), therefore we conducted a t-test to compare vane length of normal feathers against those containing pallid bands (vane length was scaled against the first-order principle-component score of overall body size extracted from each individual’s wing chord, tarsus, and head-bill lengths). To determine if there were age-related patterns in pallid band size and position, we also evaluated the pairwise correlations between all three measures (pallid band feathers only) using Pearson’s product-moment tests. All statistical analyses of feather/body morphology were conducted using the base functions available in R (R Core Team, 2014).

Observed pigmentation deficiencies in pallid bands often coincide with malformed feather barbules (Michener & Michener, 1938; Wood, 1950; Prum & Williamson, 2001; Møller, Erritzøe & Nielsen, 2009). Therefore, we examined and photographed select feathers under a dissecting microscope equipped with a digital camera. We qualitatively noted the physical attributes of barbules within pallid bands relative to: adjacent regions, normal juvenile rectrices, and feathers freshly-replaced during the late-summer post-juvenile molt that had been collected at a later date (September 11, 2013).

### Local disturbances and grasshopper sparrow breeding phenology

Specific to the May 31, 2013 weather event, we determined the probable size of hailstones that impacted our study site by consulting National Weather Service storm reports, including: ground observation reports made through the Severe Hazards Analysis & Verification Experiment (*SHAVE*; Ortega et al., 2009), and hail estimates derived from weather radar data using the Maximum Estimated Size of Hail (MESH) model (Witt et al., 1998; Stumpf, Smith & Hocker, 2004). Additionally, we examined local-scale meteorology from the ‘ELRE’ Oklahoma Mesonet station at the GRL (http://www.mesonet.org/index.php/sites/site_description/elre) to determine if there were any other weather anomalies for May–July of 2013 relative to 1999–2012 norms that may have impacted Grasshopper Sparrow foraging abilities. Finally, we interviewed GRL staff regarding the management history of the study area to determine if any anthropogenic disturbance may have occurred within that grassland paddock during the 2013 breeding season.

To determine whether the timing of the May 31 storm could have realistically affected such a large proportion of the juvenile cohort at our study site, we examined existing data on the species’ nesting phenology in Oklahoma. One of the authors (DLR) had collected these data for 149 Grasshopper Sparrow nests 200 km to the northeast in Washington and Osage Counties, Oklahoma from 1992 to 1996 as part of a separate study by the GMSARC (see Rohrbaugh et al. (1999)) for the nest searching methodology. Our preliminary analysis indicated that clutch size significantly declined over the course of the breeding season (see Fig. S1). Therefore, we restricted our use of these data to 104 nests with both final clutch size plus dates of clutch initiation (either directly observed or extrapolated based on incubation stage at discovery), and classified the clutches according to their expected status on May 31. For Grasshopper Sparrows, a typical clutch will hatch approximately 14 days after the first egg is laid and the young fledge after 11 days in the nest (Vickery, 1996). According to this schedule, by the afternoon of May 31, clutches initiated prior to May 7 would likely have fledged, clutches initiated May 7–17 would likely be nestlings, and clutches initiated May 18–31 would still be eggs (Table 1). We used a z-test implemented in R (R Core Team, 2014) to compare the proportion of juveniles captured during late August 2013 that showed synchronous pallid bands against the proportion of a typical cohort which would have been expected to have hatched prior to May 31 and were fully independent by late August.

**Table 1:** Clutch initiation phenology of 104 Grasshopper Sparrow nests at 20 sites in Washington and Osage Counties, Oklahoma. Expected status on May 31 was based upon the clutch size, and assumed incubation initiated on the penultimate egg and 11-day incubation and 11-day nestling periods (Vickery, 1996). Clutch totals represent the total number of eggs within nests that were initiated during the period indicated.

Clutch initiation period	Likely status on May 31	1992–96 clutch totals	Proportion of late-August flocked juveniles^a^	Cumulative proportion
≤ May 6	Fledged	18	4.6	4.6
May 7–17	Nestlings	83	21.1	25.6
May 18–31	Eggs	121	30.7	56.3
Jun 1–Jul 13	Pending^a^^,1^	172	43.7	100.0
>Jul 13	Pending^a^^,2^	49	n/a^a^	n/a^a^

**Notes.**

a By the late-August sampling period the surviving young produced by “pending” clutches would have been either: 1. Fully fledged and roaming. 2. Less likely to be capable of sustained flight or to have flocked with other juveniles (Vickery, 1996).

### Stable isotope analysis

To prepare feathers for analysis, we first cleaned each with dilute detergent and then a 2:1 chloroform–methanol solution (Paritte & Kelly, 2009) and dried the samples at room temperature. We then sectioned the distal end of each feather into four or five 0.25–0.40 mg portions (at least two sections grown after (i.e., proximal) and all sections preceding the pallid band (Fig. 1B)) and packed each section into a 3.5 × 5 mm tin capsule for insertion into an autosampling tray. We contained the pallid band, if present, within only one of these sections. We performed stable isotope analyses using a Thermo Delta V Plus isotope ratio mass spectrometer (Thermo Scientific, Bremen, Germany) connected to a Costech ECS 4010 elemental analyzer (Costech Analytical Technologies, Valencia, California, USA) in the Stable Isotope Laboratory at the University of Oklahoma School of Geology and Geophysics. We measured isotopic ratios for nitrogen (*δ*^15^N) and carbon (*δ*^13^C) to the nearest per mil (‰), and included as in-run standards multiple samples from the same feather of an adult Brown-headed Cowbird (*Molothrus ater*).

We used R (R Core Team, 2014) and package ‘lme4’ (Bates, Maechler & Bolker, 2012) to analyze the relationship between *δ*^15^N and, separately, *δ*^13^C relative to the presence or absence of a pallid band using generalized linear mixed models (GLMM; Bolker et al., 2009). We conducted separate GLMM analyses: (a) among groups according to pallid band presence, (b) among sections of individual feathers according to pallid band presence, and (c) among two-section regions of individual feathers that were grown during and immediately following pallid band development (i.e., the pallid band region). We included in each analysis a variable indicating the presence of a pallid band for each individual, section, or region (respectively) as the only fixed effect, and bird ID and feather section number as random effects in a random intercepts model. We examined residual plots for deviations from homoscedasticity or normality, and obtained p-values by likelihood ratio tests of each respective full model (i.e., pallid band as a fixed effect) versus its reduced/null model (i.e., without pallid band as a fixed effect; Bolker et al., 2009). Finally, we used the *partimat* function in the R package ‘klaR’ (Weihs et al., 2005) to calculate and visually represent a principal components analysis of *δ*^15^N and *δ*^13^C among feather sections grouped according to whether the source feather had a pallid band. The dividing function was based upon a linear discriminant function analysis, and an error rate was calculated based on the number of sections that were incorrectly assigned (i.e., positioned on the opposite side of the dividing line from its group mean).

## Results

### Pallid band incidence and feather characteristics

Grasshopper Sparrow juveniles captured from the GRL population displayed a very high incidence of synchronous pallid bands in the tail (11 of 25 individuals; 44%). Among the 25 juveniles examined, we noted fault bars on the wing feathers for only one individual, and these were the narrow, “true” fault bar type and were slightly asynchronous (i.e., not uniformly positioned; see Fig. S2). The pallid bands we observed among juvenile Grasshopper Sparrow rectrices were up to 3.4 mm wide, aligned across all of the tail feathers, and showed modestly-reduced pigmentation and barbule density (Figs. 1A and 3). We found that mean (±s.d.) vane length of the sampled rectrices, corrected for overall body size, did not differ between feathers having pallid bands (41.0 ± 1.1 mm) versus those sampled from normal-appearing individuals (41.0 ± 1.6 mm; *t* = 0.853, *p* = 0.406). Among feathers having pallid bands, the mean distance from band midpoint to feather tip was 8.8 ± 5.0 mm and the mean pallid band width was 1.8 ± 0.9 mm. The distance of pallid band midpoints to feather tips, as a proportion of vane length, ranged from 0.06 to 0.42 with a mean of 0.21 ± 0.12 (s.d.). Pallid band width significantly increased as a function of midpoint distance from feather tip (*r* = 0.928; *t* = 7.45; *p* < 0.001), but was not related to vane length (*r* = 0.018; *t* = 0.05; *p* = 0.958). The midpoint locations of pallid bands were likewise not correlated with vane length (*r* = − 0.025; *t* = − 0.075; *p* = 0.942). We observed that the structure of feather barbules was moderate-to-severely degraded in pallid bands, in extreme cases showing large sections of unhooked or entirely missing barbules (Fig. 3).

**Figure 2:**
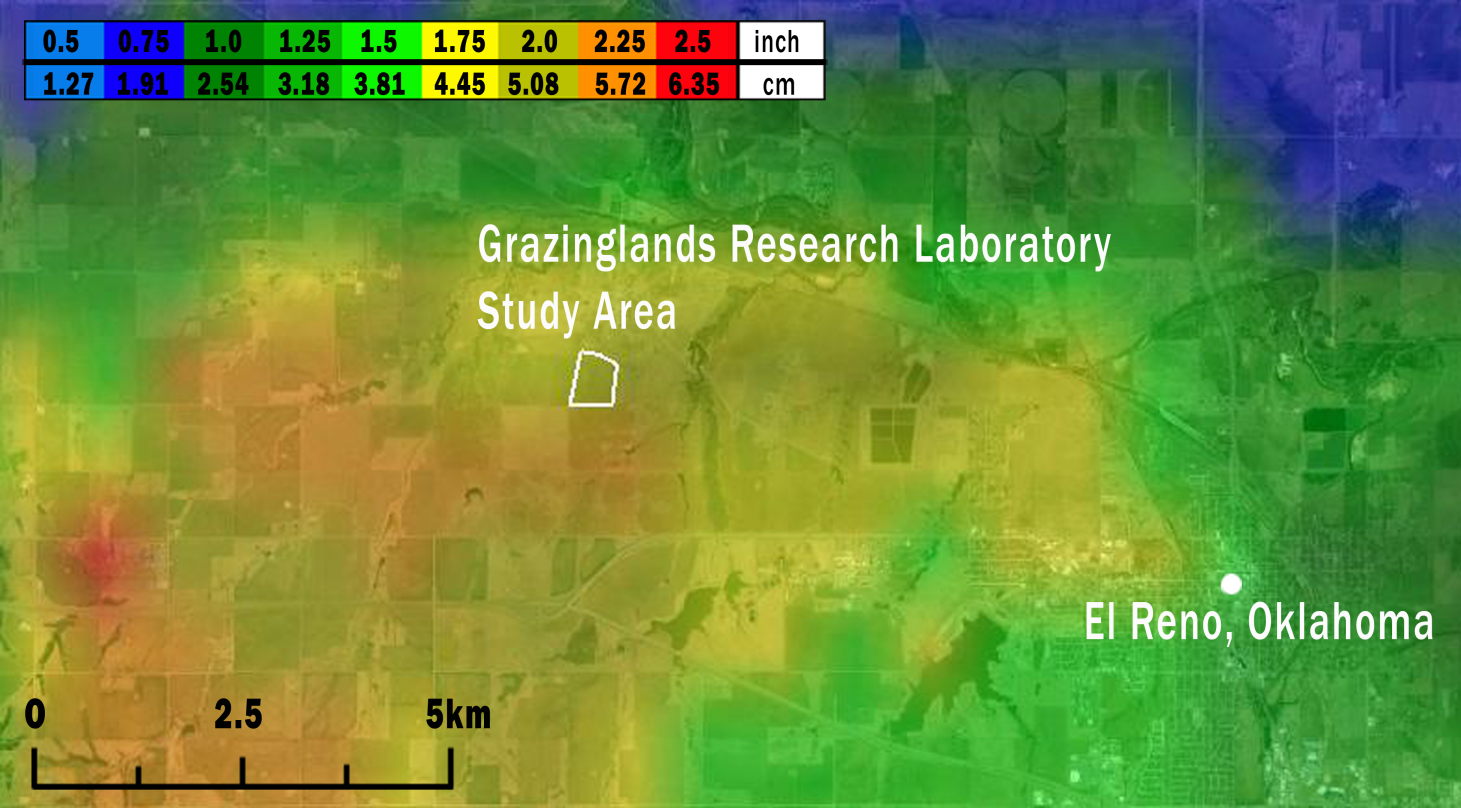
Map of the study area (white outline) showing the maximum size of hailstones that fell across the region on May 31, 2013. Hailstone size was estimated from National Weather Service WSR-88D radar data using the Maximum Estimated Size of Hail model (MESH; Witt et al., 1998; Stumpf, Smith & Hocker, 2004) and are displayed along a light blue (<1.27 cm) to red (5.72 to 6.35 cm) color scale. Image credit: the MESH archive at http://ondemand.nssl.noaa.gov/.

**Figure 3:**
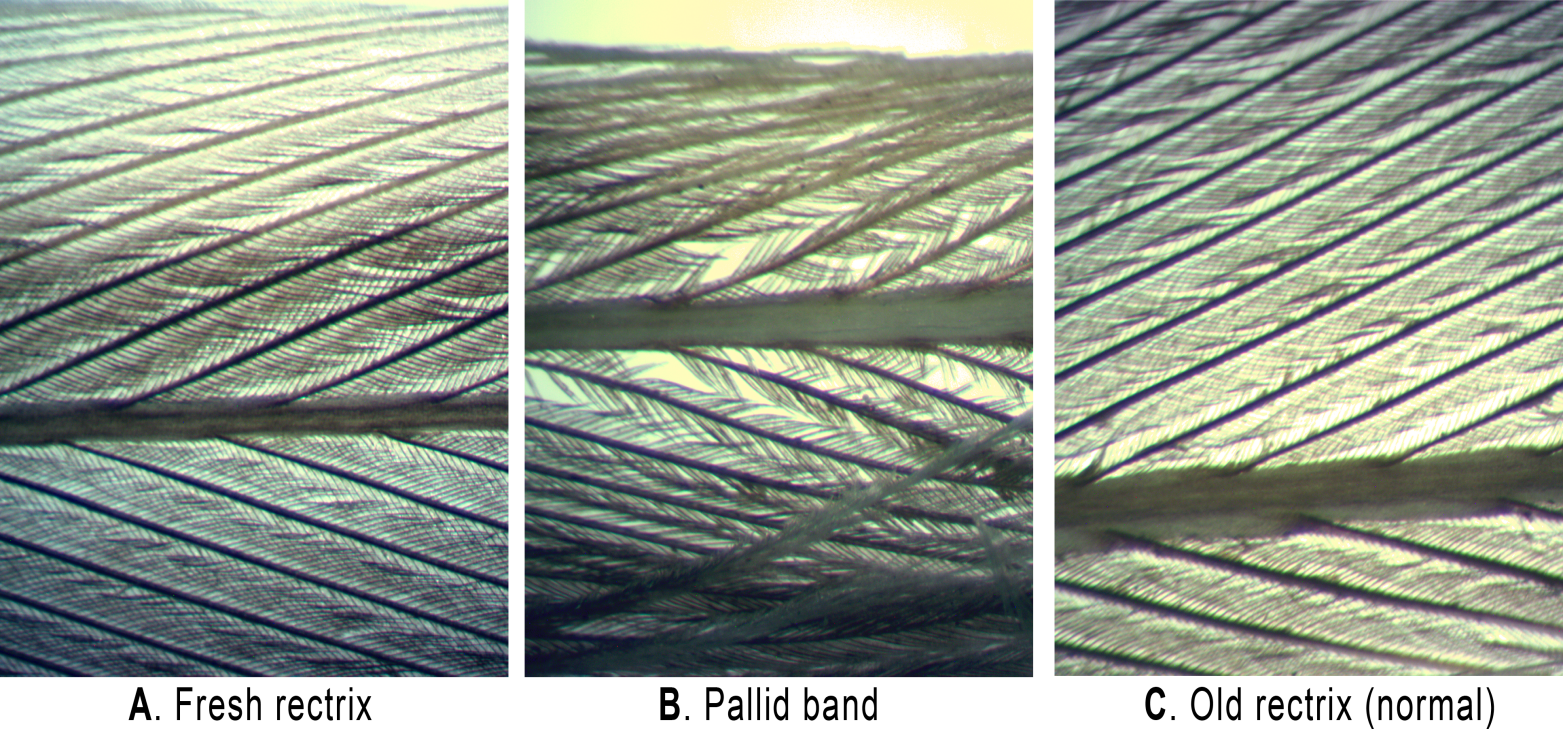
Backlit microscope views (20 ×) of Grasshopper Sparrow feather rachii and barbules. Shown are: (A) a fresh rectrix collected after post-juvenile molt, (B) a rectrix with pallid band, and (C) a normal, worn juvenile feather. Photos by Jeremy D. Ross.

### Local disturbances and breeding phenology

Based on data from both weather radar and the ‘ELRE’ Oklahoma Mesonet station at the GRL, we estimated that hail fell over the GRL during approximately 16:00–16:20 local time on May 31, 2013. Hailstone diameters estimated from weather radar data using the MESH model ranged from 4.45 to 5.72 cm at the GRL (Fig. 2). These estimates likely accurately reflect the actual hailstone sizes, since nearby ground-based observations of maximum hailstone sizes reported through *SHAVE* consistently matched or modestly exceeded the MESH estimates (see Fig. S3).

Local meteorological data also indicated that during the period when juveniles that were captured in August would have likely been in the nest or recently fledged (i.e., May 1–July 13, 2013) the amount of precipitation recorded at the GRL exceeded the site’s 1999–2012 mean by 22.4 cm, representing 150% of the normal rainfall for this period. Perhaps not surprisingly, this exceedance was primarily caused by a 12.1 cm downpour during the May 31 storm. No other daily precipitation totals exceeded 4.1 cm during this period. There were no other obvious stressors associated with the peak Grasshopper Sparrow reproductive period. At no point between May 1 and July 13, 2013 did the maximum or minimum temperature depart more than ±5 °C from 1999 to 2012 means. Other possible stressors, such as local land use and management, were similarly unlikely. During 2013, our grassland study site at the GRL was grazed lightly by cattle (∼1 per 10 ha) and the area was not managed with herbicides, pesticides, or mowing treatments during May–July (S Coleman, USDA, pers. comm., 2013)

The median date of clutch initiation observed during GMSARC surveys in Oklahoma from 1992 to 1996 was June 4, though when temporal variation in clutch size was accounted for, this median became May 31. Based on those surveys, we also estimated that among Grasshopper Sparrow offspring that would have been fully independent by late August (provided all equally hatched and survived; *n* = 394), 25.6% would have hatched or fledged and 30.7% would have been eggs in the nest as of May 31 (Table 1). The former value was significantly lower than the percentage of juveniles captured at the GRL in August 2013 showing pallid bands across their rectrices (44%; *n* = 25; *z* = − 2.03; *p* = 0.042).

### Stable isotopes

**Figure 4:**
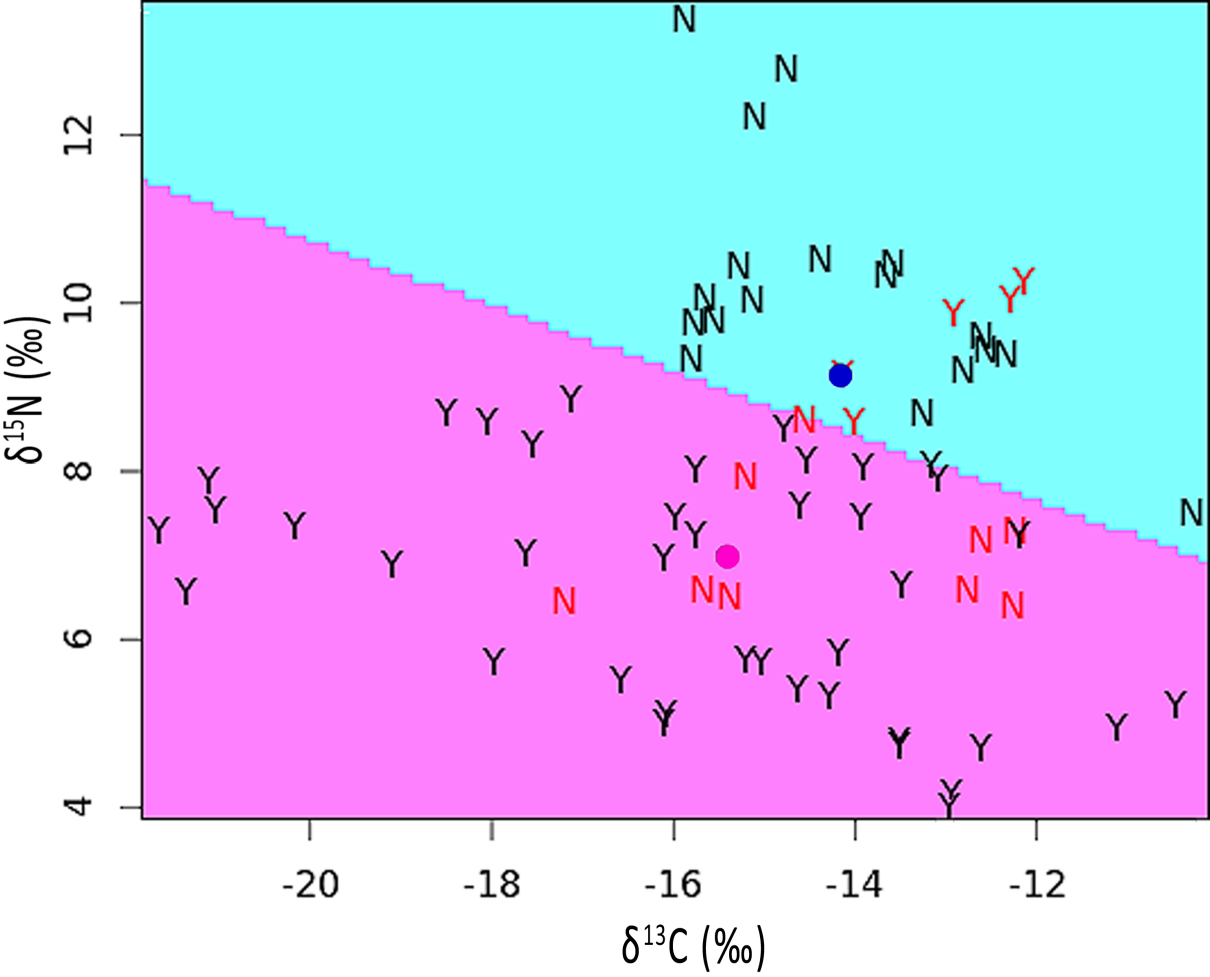
Partition plot of *δ*^15^N versus *δ*^13^C from sections analyzed within feathers containing pallid bands (Y) and normal feathers (N). The dividing line was based upon a linear discriminant function analysis. Samples indicated in red font signify misassignments (19.4% of sections). Colored dots represent the group means for feathers with pallid bands (pink) or normal feathers (blue).

The mean stable isotope ratios in feathers showing pallid bands were 2.2‰ lower with respect to *δ*^15^N but 1.2‰ higher for *δ*^13^C when compared to normal appearing feathers (see Table S1). After correcting for variation among individuals and feather sections, the difference was significant for *δ*^15^N [*χ*^2^(1) = 5.90, *p* = 0.015] but not for *δ*^13^C [*χ*^2^(1) = 1.55, *p* = 0.213]. The discriminant function analysis partition plot indicated that only 19.4% of sections were misassigned relative to their true group (pallid band or normal; Fig. 4).

Sections containing pallid bands did not differ from other parts of the feather for either *δ*^15^N [*χ*^2^(1) = 1.10, *p* = 0.295] or *δ*^13^C [*χ*^2^(1) = 0.35, *p* = 0.553]. However, if we paired data from sections containing pallid bands with those subsequently grown (i.e., immediately proximate), then relative to the remaining sections, this pallid band region contained significantly elevated *δ*^15^N [*χ*^2^(1) = 4.81, *p* = 0.028], while *δ*^13^C remained undifferentiated [*χ*^2^(1) = 1.96, *p* = 0.162]. On average, the presence of a pallid band was associated with an increase in *δ*^15^N of about 0.37 ± 0.16 (S.E.)‰, which falls well above the 0.09‰ error rate calculated from independent runs of our Brown-headed Cowbird feather standard.

## Discussion

Our findings are all consistent with pallid bands among juvenile Grasshopper Sparrows at GRL likely resulting from an intense local stressor, and the most likely cause was the May 31, 2013 severe storm that impacted the region with tornadoes, damaging winds, and large hail (Uccellini, 2014). Beyond this simple event attribution, our findings provide some of the first evidence about the sublethal impacts of severe weather and how native bird species may be affected by such periodic stressors.

### Stable isotopes as records of environmental stressors

Nitrogen isotopes within the pallid band region were significantly enriched in ^15^N relative to other parts of the same feathers. These spikes in *δ*^15^N support our predictions and are consistent with increased muscle catabolism as part of the stress response that produced the pallid bands. There was also notable among-individual variation both in the slope and magnitude of *δ*^15^N and *δ*^13^C along the feather vane, which suggested differences in diets during development consistent with young originating from different nests. This lends confidence that the sampled individuals suitably represented the population variation.

The magnitude of differences between feathers from juveniles possessing normal feathers versus juveniles displaying pallid bands for both *δ*^13^C (−1.2‰) and *δ*^15^N (2.2‰) would be consistent with trophic or successional (i.e., C4 to C3 plant community) shifts that one might expect among individuals growing feathers at different times during a temperate breeding season (Kelly, 2000). More specifically, if we assume that juveniles without pallid bands were reared after the large scale disturbance, then the data are consistent with a scenario in which the diets provisioned to Grasshopper Sparrow nestlings and fledglings shifted as the season progressed. Increased *δ*^15^N in sampled rectrices of normal appearance could be attributed to ingestion of insect prey items from higher trophic levels (e.g., larger, omnivorous grasshoppers), and the difference in *δ*^13^C could result from an increasingly C3 plant base (Hobson, 1999; Kelly, 2000; West et al., 2006). This trophic progression may even be evident within individual feathers where *δ*^15^N naturally increases from tip-to-root, as observed in this study and by Symes & Woodborne (2011) in White-bellied Sunbirds (*Cinnyris talatala*).

### Pallid bands as a response to stress

High rates of fault bars have been reported in other species (Hawfield, 1986; Bortolotti, Dawson & Murza, 2002; Møller, Erritzøe & Nielsen, 2009), but such accounts appear entirely restricted to the narrow (i.e., ≤1 mm) “true” fault bars that we earlier distinguished from pallid bands. The incidence of pallid bands within a population appears to be much rarer (Michener & Michener, 1938; Erritzøe & Busching, 2006). In fact, only 1.5% of 271 Grasshopper Sparrow juveniles captured at 22 other sites in Nebraska, Kansas, and Oklahoma during 2013–14 had pallid bands similar to those observed in such prevalence at the GRL in 2013 (WA Boyle, 2013–14, unpublished data). The underlying cause of the high incidence of pallid bands at the GRL was, therefore, likely a rare event that was sufficiently intense and widespread to affect much, if not all, of the local Grasshopper Sparrow breeding population; most probably the severe weather outbreak of May 31, 2013.

The emergence of pallid bands appears to follow a strong linear pattern between position along the length of the feather and width. If pallid bands in our study population all represented the same period of growth, then this pattern would indicate that feather growth linearly increased as feathers became longer. However, Elderbrock, Kern & Lynn (2012) found that although width of growth bars down the vane of individual feathers in juvenile Eastern Bluebirds (*Sialia sialis*) did vary substantially, these differences were randomly located and did not linearly increase with position down the feather vane. Instead, the authors noted that growth bar width was disconnected from the rate of feather elongation, which was constant during the development of individual feathers. Rather than feather growth increasing with age, our observed patterns in pallid band width relative to distance from the tip more likely indicate that the duration of the stress response had linearly increased as a function of chick age. This inference agrees with prior studies showing that older chicks are more prone to an extended stress response due to increased development of the hypothalamic-pituitary-adrenal axis (Sims & Holberton, 2000; Sockman & Schwabl, 2001; Wada, Hahn & Breuner, 2007). Moreover, the increasingly-independent fledglings are regressively provisioned by the adults while simultaneously experiencing greater metabolic demands and, therefore, are usually closer to starvation than younger chicks (Blem, 1975). In at least three prior studies of age-related patterns and true fault bars this relationship was reversed (i.e., older chicks displayed fewer fault bars; Machmer et al., 1992; Jovani & Tella, 2004; Jovani & Diaz-Real, 2012) again suggesting that the two types of fault bars fundamentally differ in causation.

### Ecological and evolutionary implications of pallid bands

The narrow and abrupt feather malformations we previously referred to as “true” fault bars are prone to breakage (Sarasola & Jovani, 2006), which affects flight performance (Murphy, Miller & King, 1989; Norberg, 1990; Jovani et al., 2010) and may explain why Goshawk (*Accipiter gentilis*) prey had significantly higher-than-average incidences of fault bars relative to a random sample of the same species from the same general area (Møller, Erritzøe & Nielsen, 2009). Jovani & Blas (2004) argued that, in light of this, true fault bars should be less likely to occur among feather tracts that are strongly tied to flight performance, because evolutionary pressures would have favored individuals that were capable of inherently allocating greater resources to feathers critical for flight (i.e., the “fault bar allocation hypothesis”). Though pallid bands may be less likely to represent a breakage risk, they do contain structural weaknesses that could affect flight performance and durability (Fig. 3). Among the Grasshopper Sparrows examined in this study, the appearance of pallid bars exclusively in the rectrices with only one example of true fault bars in the remiges (see Fig. S2) suggests that the fault bar allocation hypothesis may apply to pallid bars as well, and is worthy of further investigation. It may be the case that ground nesting birds like Grasshopper Sparrows, which are frequently exposed to environmental stressors (Nice, 1957; Ricklefs, 1969), would benefit by coupling fault bar/pallid band allocation with their complete late-summer post-juvenile molt (Pyle, Jones & Ruth, 2008) as an adaptive response to stress during early development (Pap et al., 2007).

### Biological relevance of severe weather

Local environmental conditions dictate whether species survive and reproduce successfully in any given area. Lack (1966) suggested that extremes in local environments would be strongly responsible for limiting species, as these would present the most grievous of stressors. Severe weather is a prime example, as it can cause widespread mortality and is known to necessitate specific local adaptations within biological communities (Wingfield, 1988; Newton, 2007). Severe weather impacts on birds have generally been studied in association with large, widespread, and relatively long-lasting events such as cold snaps, hurricanes, blizzards, and regional storm fronts (Whitmore, Mosher & Frost, 1977; Wiley & Wunderle, 1993; Brown & Brown, 1998; Newton, 2007; Frederiksen et al., 2008; Rittenhouse et al., 2010; Streby et al., 2015). However, intense but relatively localized perturbations, such as large hail or tornadoes associated with severe thunderstorms, have primarily received attention only after the most obvious and widespread destruction (e.g., Gates, 1933; McClure, 1945; Smith & Webster, 1955; Narwade et al., 2014). Yet, severe thunderstorms can vary in their degree of impact across species and, thus, may represent a particularly relevant factor in regulating biological community composition. Considering that much of the American Great Plains experiences severe thunderstorms and hail annually (Doswell, Brooks & Kay, 2005; Cecil & Blankenship, 2012; Cintineo et al., 2012) and that hailstones as small as 1 cm diameter can destroy eggs and injure adult birds (Hanford, 1913; Graul, 1975; Hume, 1986; Narwade et al., 2014), these weather events are likely to have profound ecological and conservation relevance to grassland species, especially ground-nesting birds. This is especially true during the key breeding period of April–July, when vulnerable adults, eggs, and young face the peak of the severe thunderstorm season.

In our study, Grasshopper Sparrow juveniles with ‘normal’ rectrices appear to show the stable isotope signatures of hatching after May 31. Our analysis indicated that such individuals were significantly less abundant than expected based on the species’ nesting phenology in Oklahoma (i.e., 56% in 2013 versus 74.4% in 1992–96). This could be due to slight climatic differences between study areas, such as the date of last spring freeze (Oklahoma Climatological Survey, 2014), or because of 20-years of climate change. Alternatively, the lower proportion of late-hatching young in 2013 could have reflected an actual net loss among the latter half of the 2013 cohort, in this case ∼25% relative to the 1992–96 demography. It is entirely reasonable to expect that hailstones which exceeded 4 cm in diameter could have led to widespread destruction of eggs (an estimated 30% of a typical cohort on May 31), or that this stressor could have disrupted late-season nesting attempts (e.g., adults directly killed or abandoned the area after the storm).

Scientifically assessing the biological impacts of severe weather might be viewed as being limited by the ability to predict in advance where storms will strike, precluding before-and-after comparisons. However, every year there are likely field studies occurring at points throughout regions where severe weather is likely to strike (Doswell, Brooks & Kay, 2005; Cecil & Blankenship, 2012; Cintineo et al., 2012) and, so, a broadly-distributed network of researchers could readily be assembled to study large-scale ecological consequences of severe weather. We call for the ecological research community to take advantage of severe storm events as they occur at their research sites, opportunistically sampling biologically informative data required to assess the nature and magnitude of such stressors on animal communities. In the face of a changing climate and the expected shifts in severe weather regimes (Trapp et al., 2007; Goodess, 2013) there is a need to expand our knowledge of the current and future ecological impacts of severe weather events (Jentsch, Kreyling & Beierkuhnlein, 2007), so that we may work toward mitigating losses among vulnerable species and biological communities.

## Supplemental Information

10.7717/peerj.814/supp-1 Table S1 Nitrogen and carbon stable isotope fractionation (‰) within sections of juvenile Grasshopper Sparrow rectrices Sections were sequential samples of approximately equal weight taken from the tip (Section 1) proximally. Sections containing fault bars are highlighted with grey boxes. In three instances, the section sample was lost due to an error during the stable isotope analysis process (indicated as “*Lost”*).

10.7717/peerj.814/supp-2 Figure S1 Seasonal trend in Grasshopper Sparrow clutch size in Oklahoma The data are based on surveys during 1992–96 and include only those clutches known to have been completed (i.e., reached incubation stage). The linear fit (*y* = − 0.0152*x* + 6.72 based on Julian dates) has a significantly non-zero slope (*R*^2^ = 0.192, *t* = − 4.93, *d*.*f*. = 102, *p* < 0.001).

10.7717/peerj.814/supp-3 Figure S2 Photograph of true fault bars in the primary wing feathers of a juvenile Grasshopper Sparrow Note that compared to the pallid bands seen in the rectrices, these fault bars are narrower, do not show a loss in pigmentation, and are not aligned across feathers. The tail of the same individual is pictured in Fig. 1A.

10.7717/peerj.814/supp-4 Figure S3 Maximum hailstones during May 31, 2013 storm estimated using MESH analysis of radar data or from telephone surveys of local observers (*SHAVE*) Surveyed locations were within 30 km of the Grazinglands Research Laboratory. A dotted line indicates parity between the two estimates. A linear regression through the observations is presented as a solid line (equation and *R*^2^ indicated).

## Additional Information and Declarations

### Field Study Permissions

The following information was supplied relating to field study approvals (i.e., approving body and any reference numbers): Banding and feather sampling was conducted with permission to WA Boyle from the USFWS (permit #23836) and from the state of Oklahoma’s Wildlife Conservation Department (permit #5762).
