# Supplementary material for: Pallid bands in feathers and associated stable isotope signatures reveal effects of severe weather stressors on fledgling sparrows
Source: PeerJ. 2015 Mar 3;3:e814. doi: 10.7717/peerj.814 (PMC4358640; doi:10.7717/peerj.814)
Supplement: Table S1 — Sections were sequential samples of approximately equal weight taken from the tip (Section 1) proximally. Sections containing fault bars are highlighted with grey boxes. In three instances, the section sample was lost due to an error during the stable isotope analysis process (indicated as “Lost”). [file peerj-03-814-s001.docx]

| Individual (Band Number) | Fault Bar | Section 1 | | Section 2 | | Section 3 | | Section 4 | | Section 5 | | All Sections (mean±s.d.) | |
| --- | --- | --- | --- | --- | --- | --- | --- | --- | --- | --- | --- | --- | --- |
|  |  | δ^15^N | δ^13^C | δ^15^N | δ^13^C | δ^15^N | δ^13^C | δ^15^N | δ^13^C | δ^15^N | δ^13^C | δ^15^N | δ^13^C |
| 1831-10723 | No | 9.71 | -15.85 | 12.15 | -15.18 | 12.73 | -14.84 | 13.36 | -15.95 |  |  | 11.98±1.60 | -15.45±0.53 |
| 1831-10725 | No | 9.98 | -15.21 | 10.46 | -14.47 | 10.27 | -13.73 | 10.40 | -13.65 |  |  | 10.28±0.21 | -14.26±0.73 |
| 1831-10736 | No | 9.72 | -15.64 | 10.01 | -15.74 | 10.37 | -15.35 | 9.27 | -15.87 |  |  | 9.84±0.47 | -15.65±0.22 |
| 1831-10742 | No | 7.87 | -15.29 | *Lost* | *Lost* | 9.14 | -12.88 | 8.55 | -14.63 |  |  | 8.52±0.63 | -14.27±1.25 |
| 1831-10743 | No | 6.51 | -12.83 | 7.21 | -12.31 | 7.44 | -10.33 | 7.11 | -12.70 |  |  | 7.07±0.39 | -12.04±1.16 |
| 1831-10745 | No | 6.39 | -17.28 | 6.53 | -15.75 | 6.33 | -12.34 | 6.43 | -15.46 |  |  | 6.42±0.09 | -15.21±2.07 |
| 1831-10747 | No | 8.63 | -13.33 | 9.33 | -12.42 | 9.38 | -12.65 | 9.55 | -12.70 |  |  | 9.22±0.40 | -12.78±0.39 |
| Normal Feather: | Mean | 8.40 | -15.06 | 9.28 | -14.31 | 9.38 | -13.16 | 9.24 | -14.42 |  |  | 9.07±1.94 | -14.24±1.62 |
|  | s.d. | 1.52 | 1.52 | 2.10 | 1.58 | 2.09 | 1.68 | 2.28 | 1.42 |  |  |  |  |
| 1831-10714 | Yes | 7.17 | -15.83 | 8.24 | -17.62 | 8.52 | -18.13 | 8.78 | -17.19 |  |  | 8.18±0.71 | -17.19±0.99 |
| 1831-10718 | Yes | 7.26 | -20.25 | 6.81 | -19.17 | 7.95 | -15.82 | 7.51 | -14.69 | 6.90 | -16.17 | 7.29±0.47 | -17.22±2.37 |
| 1831-10722 | Yes | 7.97 | -13.99 | 8.01 | -13.23 | 7.85 | -13.16 | 7.17 | -12.26 |  |  | 7.75±0.39 | -13.16±0.71 |
| 1831-10724 | Yes | 8.61 | -18.58 | 8.05 | -14.60 | 8.50 | -14.09 | 7.38 | -14.00 |  |  | 8.13±0.56 | -15.32±2.19 |
| 1831-10726 | Yes | 5.65 | -15.11 | 5.03 | -16.16 | 5.43 | -16.65 | 5.66 | -18.05 |  |  | 5.44±0.30 | -16.49±1.22 |
| 1831-10734 | Yes | 6.96 | -17.70 | 7.37 | -16.05 | 8.42 | -14.87 | *Lost* | *Lost* |  |  | 7.58±0.75 | -16.21±1.42 |
| 1831-10735 | Yes | 6.48 | -21.44 | 7.80 | -21.20 | 7.22 | -21.79 | 7.46 | -21.12 |  |  | 7.24±0.56 | -21.39±0.30 |
| 1831-10737 | Yes | 9.09 | -14.22 | 10.19 | -12.22 | 9.98 | -12.37 | 9.81 | -13.00 |  |  | 9.77±0.48 | -12.95±0.91 |
| 1831-10738 | Yes | 4.92 | -16.17 | *Lost* | *Lost* | 6.57 | -13.57 | 4.71 | -13.58 | 4.64 | -12.68 | 5.21±0.91 | -14.00±1.50 |
| 1831-10748 | Yes | 5.34 | -14.72 | 5.67 | -15.29 | 5.24 | -14.36 | 5.76 | -14.27 |  |  | 5.50±0.25 | -14.66±0.46 |
| 1831-10749 | Yes | 3.92 | -13.03 | 4.09 | -13.02 | 5.14 | -10.54 | 4.88 | -11.19 | 4.66 | -13.58 | 4.54±0.52 | -12.27±1.32 |
| Fault bar feather: | Mean | 6.67 | -16.46 | 7.13 | -15.85 | 7.35 | -15.03 | 6.91 | -14.94 | 5.40 | -14.14 | 6.91±1.63 | -15.48±2.82 |
|  | s.d. | 1.60 | 2.71 | 1.78 | 2.84 | 1.58 | 3.05 | 1.66 | 3.01 | 1.30 | 1.81 |  |  |
| All Individuals: | Mean | 7.34 | -15.91 | 7.93 | -15.27 | 8.14 | -14.30 | 7.87 | -14.72 | 5.40 | -14.14 | 7.72±2.03 | -15.01±2.50 |
|  | s.d. | 1.75 | 2.37 | 2.13 | 2.51 | 2.01 | 2.71 | 2.21 | 2.43 | 1.30 | 1.81 |  |  |
