# Supplementary figures and images for: Pallid bands in feathers and associated stable isotope signatures reveal effects of severe weather stressors on fledgling sparrows
Source: PeerJ. 2015 Mar 3;3:e814. doi: 10.7717/peerj.814 (PMC4358640; doi:10.7717/peerj.814)

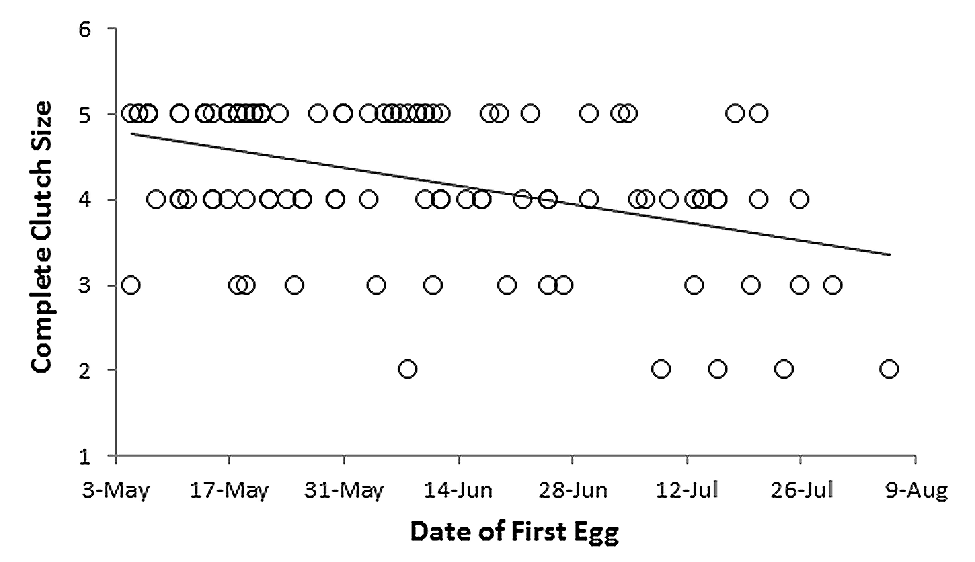

Supplement: Figure S1 — The data are based on surveys during 1992–96 and include only those clutches known to have been completed (i.e., reached incubation stage). The linear fit (y = − 0.0152x + 6.72 based on Julian dates) has a significantly non-zero slope (R2 = 0.192, t = − 4.93, d.f. = 102, p < 0.001). [file peerj-03-814-s002.png]

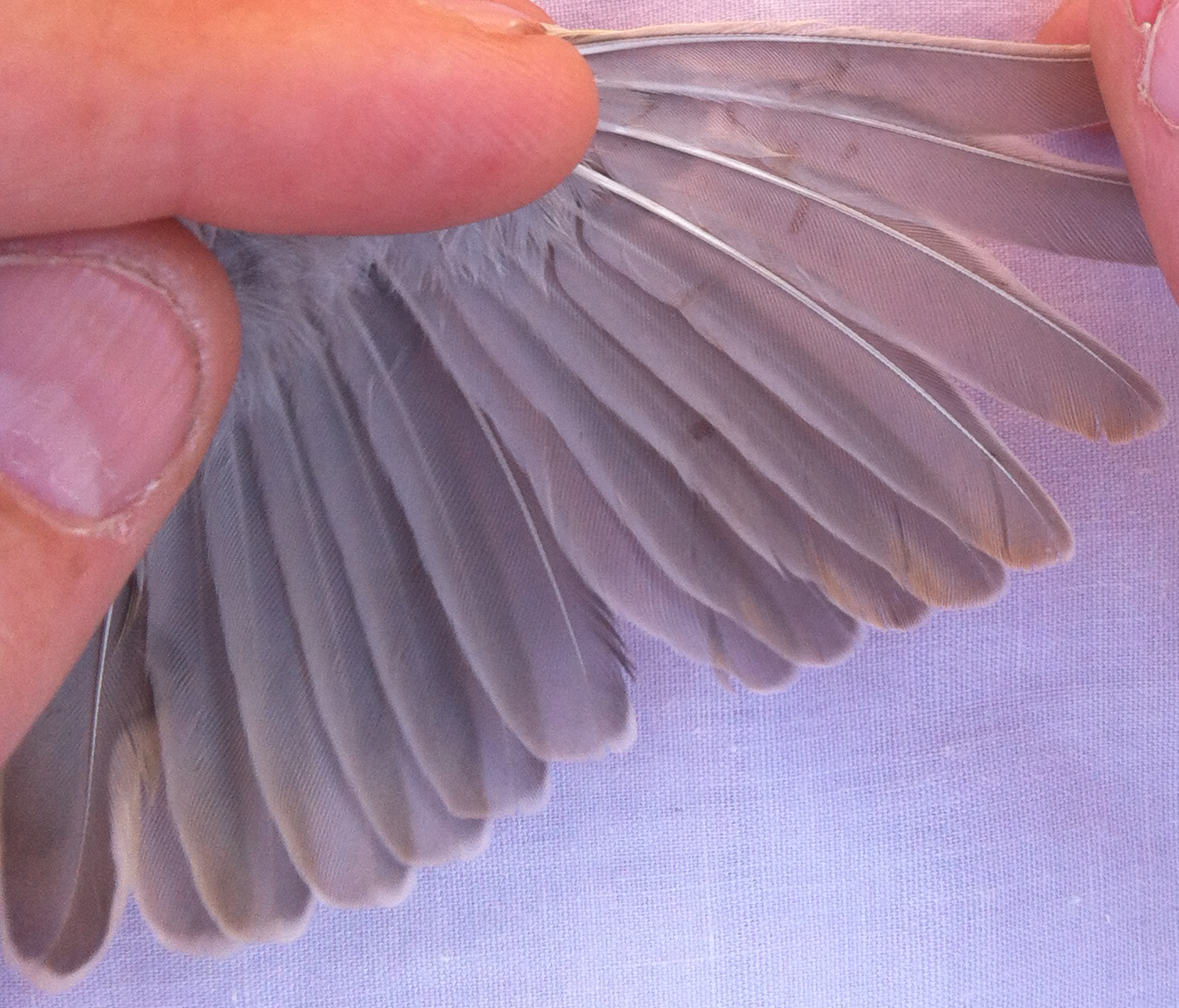

Supplement: Figure S2 — Note that compared to the pallid bands seen in the rectrices, these fault bars are narrower, do not show a loss in pigmentation, and are not aligned across feathers. The tail of the same individual is pictured in Fig. 1A. [file peerj-03-814-s003.png]

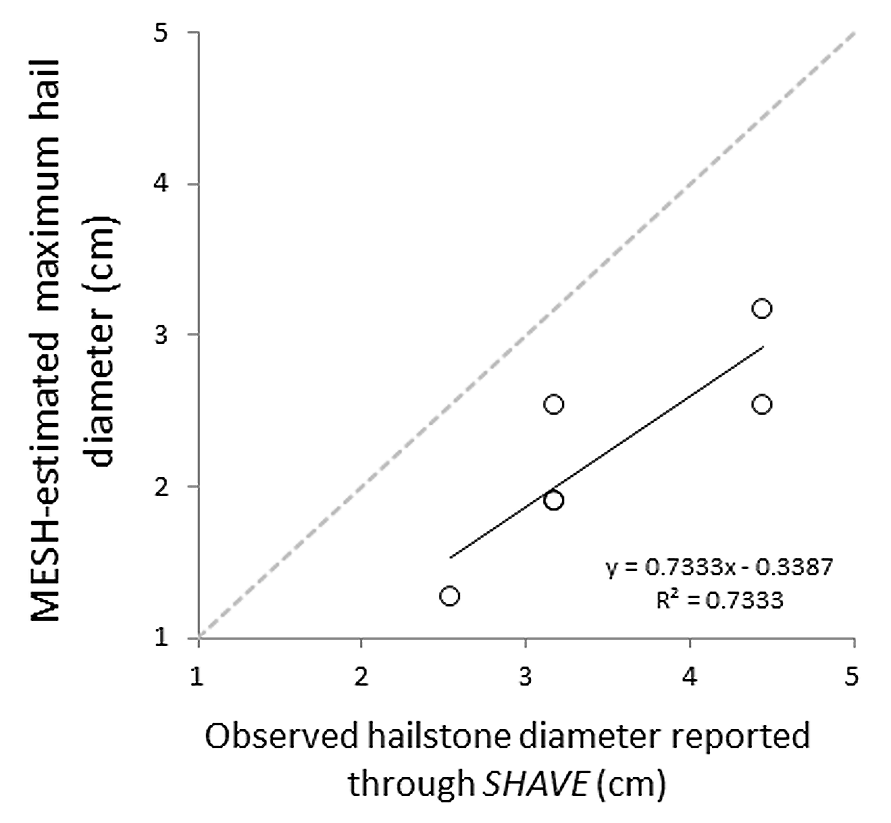

Supplement: Figure S3 — Surveyed locations were within 30 km of the Grazinglands Research Laboratory. A dotted line indicates parity between the two estimates. A linear regression through the observations is presented as a solid line (equation and R2 indicated). [file peerj-03-814-s004.png]
